# Artificial intelligence-based quantification of retinal microvascular biomarkers from fundus photography of chronic kidney disease: a case-control study

**DOI:** 10.3389/fmed.2026.1719984

**Published:** 2026-05-18

**Authors:** Qiumei Gu, Min Liu, Weiwei Zhang, Zhengju Chen, Xingye Wang, Jie Wang, Ziyan He, Fang Lu

**Affiliations:** 1 Department of Ophthalmology, West China Hospital, Sichuan University, Chengdu, China; 2 Department of Nephrology, West China Hospital, Sichuan University, Chengdu, China; 3 West China School of Nursing, Sichuan University, Chengdu, China; 4 Department of Radiology, Huaxi MR Research Center (HMRRC), Institute of Radiology and Medical Imaging, West China Hospital, Sichuan University, Chengdu, China; 5 EVision Technology (Beijing) Co., Ltd., Beijing, China

**Keywords:** artificial intelligence, chronic kidney disease, fundus photography, retinal biomarkers, microvascular parameters

## Abstract

**Background:**

Chronic kidney disease (CKD) is frequently asymptomatic in its early stages and remains substantially underdiagnosed, largely due to the lack of accessible and non-invasive risk identification tools. Retinal microvascular alterations may reflect systemic microvascular changes associated with CKD, offering a potential non-invasive window for early disease-related microvascular assessment.

**Objective:**

To identify retinal microvascular parameters associated with CKD and evaluate their discriminatory ability using AI-based fundus image analysis.

**Methods:**

In this single-center case-control study, fundus photographs from healthy controls and patients with CKD were analyzed using an AI-based platform to quantify retinal vascular features. To avoid inter-eye dependency, one eye per participant was included. A total of 322 participants were analyzed, including 110 controls, 142 with CKD stages 1–2, and 70 with CKD stages 3–5. Feature selection was performed using LASSO regression, followed by multivariable logistic regression adjusted for age, sex, and body mass index (BMI). Model performance was evaluated using receiver operating characteristic (ROC) analysis.

**Results:**

In multivariable analysis adjusted for age, sex, and BMI, lower arteriovenous ratio (AVR; OR = 0.617, *P* = 0.006), reduced arterial tortuosity (aTort, OR = 0.380, *P* < 0.001), and lower arterial vascular density (aVD, OR = 0.642, *P* = 0.027) were associated with CKD, whereas higher venous vascular density (vVD, OR = 1.910, *P* < 0.001) and higher vessel tortuosity within the 3PD region (VT3PD, OR = 2.020, *P* = 0.012) showed positive associations. The final model demonstrated moderate discrimination for CKD (AUC = 0.776). In exploratory analysis, discrimination between early and advanced CKD was limited (AUC = 0.748).

**Conclusion:**

A limited set of AI-derived retinal microvascular parameters was associated with CKD and demonstrated moderate discriminatory ability. Notably, several retinal parameters were already altered in early-stage CKD, with consistent directional changes observed across disease stages. These findings suggest that retinal microvascular alterations may be detectable in the early stages of CKD, highlighting their potential as non-invasive indicators of early disease-related microvascular changes. Further validation in larger and more diverse populations is warranted.

## 1 Introduction

Chronic kidney disease (CKD) poses a substantial global health burden, affecting more than 10% of the general population worldwide. Its prevalence continues to rise alongside increasing rates of diabetes, hypertension, and obesity–major drivers of disease development (1, 2). CKD is frequently asymptomatic in its early stages and remains underdiagnosed, contributing to significant morbidity and mortality worldwide and projected to become one of the leading causes of death by 2050 (3, 4). The burden of CKD is further exacerbated by suboptimal screening practices and limited disease awareness, particularly among high-risk populations such as older adults and individuals with comorbidities (5–7). These challenges highlight the need for accessible and non-invasive approaches for CKD risk identification. Contemporary renal biomarkers, including serum creatinine, cystatin C, and proteinuria, have limitations in capturing early microvascular alterations associated with CKD (8). While circulating and genetic biomarkers show promise for improving CKD detection and risk prediction, tissue-derived biomarkers–which reflect cumulative damage from vascular injury and other non-traditional risk factors–are generally unsuitable for non-invasive assessment. Retinal image-derived quantitative biomarkers have emerged as promising tools for early CKD screening. For instance, the deep learning model UWF-CKDS utilizes ultra-wide-field fundus images to predict CKD by extracting retinal vessel and microvascular features, and has been validated across multiple clinical institutions (9, 10).

Retinal imaging has emerged as a non-invasive modality for assessing systemic microvascular status. Several studies have reported associations between retinal vascular features and renal function, suggesting that retinal microvascular alterations may reflect systemic vascular changes related to CKD (11, 12). In this context, artificial intelligence–based analysis of fundus images enables standardized and reproducible quantification of retinal vascular features (13).

Fundus photography, as a widely accessible retinal imaging modality, enables quantitative assessment of retinal microvascular parameters, such as arteriovenous ratio (AVR), vascular density (VD), tortuosity, and fractal dimension (FD), which have been associated with systemic microvascular alterations in CKD (14). Compared with conventional renal biomarkers, retinal image–derived parameters offer advantages including non-invasiveness and the ability to capture microvascular changes in a standardized manner (15). Despite growing interest in retinal imaging biomarkers for kidney disease, several methodological issues remain insufficiently addressed. Prior studies have often included a large number of features without clear prioritization, while inter-eye dependency and confounding factors have not always been adequately accounted for (16). Most studies have focused on stages 3–5 of chronic kidney disease (estimated glomerular filtration rate < 60 ml/min/1.73 m^2^) (17). In addition, the relative contribution of individual retinal parameters remains unclear. Therefore, the present study aimed to identify a limited set of retinal microvascular parameters that differ between CKD and non-CKD individuals and remain associated with CKD after adjustment for key confounders, with a focus on clinically interpretable and potentially translatable biomarkers (18).

## 2 Materials and methods

### 2.1 Study population

This was a single-center case-control study conducted at West China Hospital, Sichuan University. Healthy controls and patients with CKD were consecutively recruited between October 15, 2024, and July 1, 2025. In the original cohort, 111 healthy controls and 229 patients with CKD were enrolled. To address inter-eye dependency, the primary analysis was restricted to one eye per participant. The right eye was selected by default; if image quality was insufficient, the left eye was used. A total of 322 participants were included in the final single-eye analysis, including 110 controls, 142 participants with CKD stages 1–2, and 70 participants with CKD stages 3–5. All participants underwent clinical evaluation, including demographic characteristics, medical history, medication use, and anthropometric measurements. CKD stage was determined by nephrologists according to the KDIGO classification based on eGFR and albuminuria. The study was conducted in accordance with the Declaration of Helsinki and approved by the Ethics Committee of West China Hospital, Sichuan University (Approval No. 20241693). It was registered with the Chinese Clinical Trial Registry (Registration No. ChiCTR2400090922), and written informed consent was obtained from all participants.

### 2.2 Inclusion and exclusion criteria

Eligible participants were adults (≥18 years). CKD was defined according to KDIGO criteria as abnormalities of kidney structure or function present for at least 3 months, including either reduced eGFR (<60 mL/min/1.73 m^2^) or markers of kidney damage such as albuminuria. Participants with CKD were categorized according to KDIGO eGFR stages: stages 1–2 (eGFR ≥ 60 mL/min/1.73 m^2^ with evidence of kidney damage), stage 3 (eGFR 30–59 mL/min/1.73 m^2^), and stages 4–5 (eGFR ≤ 29 mL/min/1.73 m^2^, corresponding to G4–G5). For the primary grouped analysis, stages 3–5 were combined to improve statistical stability due to limited sample size in advanced CKD stages.

Exclusion criteria included: a history of acute kidney injury; renal replacement therapy (dialysis or transplantation); ocular conditions that could affect retinal image analysis (e.g., non-diabetic retinopathy, severe cataract, or media opacities precluding adequate image quality); uncontrolled hypertension (systolic blood pressure > 180 mmHg or diastolic blood pressure > 110 mmHg); active systemic infections; malignancy; pregnancy; or inability to comply with study procedures.

Chronic kidney disease staging was confirmed by nephrologists, and these stage groupings were used for subsequent analyses.

### 2.3 Image quality control and fundus image acquisition

Image quality control was performed prior to quantitative analysis to ensure reliable vascular segmentation. Fundus photographs were obtained using a non-mydriatic fundus camera (Clarus 500; Carl Zeiss Meditec, Inc., Singapore) with a 133° field of view centered on the macula. Images were acquired under standardized illumination conditions, without pharmacologic pupil dilation unless required for adequate visualization.

Image quality was assessed based on predefined criteria, including focus, illumination, field definition, and vessel visibility. Images were classified as gradable or ungradable. Images were excluded if severe media opacity, poor focus, inadequate illumination, substantial motion artifacts, or incomplete retinal field coverage prevented reliable segmentation. For participants with borderline image quality, both eyes were reviewed and the eye with better analyzability was selected for the primary single-eye analysis. Only gradable images were included in the final analysis. Although a formal quantitative image quality score was not available, all images underwent standardized visual quality assessment prior to analysis. In addition, analyses restricted to images with clearly adequate quality yielded consistent results, supporting the robustness of the findings.

### 2.4 AI-enabled retinal image processing and parameter quantification

Retinal fundus images were analyzed using EVisionAI software (version 4.0; EVision Technology, Beijing, China Co., Ltd.,) (19, 20), an artificial intelligence–based platform integrating computer vision and deep learning algorithms for automated retinal image analysis. The analysis pipeline included image preprocessing, vessel and optic disk segmentation, and quantitative feature extraction. Preprocessing steps included region-of-interest extraction, normalization, denoising, and contrast enhancement to improve image quality and reduce variability. Automated segmentation of the retinal vasculature and optic disk was performed using deep learning–based algorithms. Segmentation outputs were subsequently reviewed visually, and images with failed or unreliable segmentation were excluded to ensure measurement quality.

A predefined set of retinal vascular parameters was quantified, including AVR, FD, vVD, aVD and tortuosity-related measures. These parameters were selected based on prior literature and their biological relevance to systemic microvascular alterations. All measurements were generated automatically by the software without manual intervention. To enhance reproducibility, all outputs were reviewed for plausibility prior to statistical analysis, and cases with segmentation failure were excluded from the final dataset. Only images with reliable segmentation were included in the analysis. The analysis was performed in a blinded manner with respect to clinical data.

### 2.5 Statistical analysis

Statistical analyses were performed using R software (version 4.5.1; R Foundation for Statistical Computing, Vienna, Austria). To avoid inter-eye correlation, the primary analysis was restricted to one eye per participant. Continuous variables are presented as mean ± standard deviation, and categorical variables as counts and percentages. Group comparisons were performed using analysis of variance or the Kruskal–Wallis test, as appropriate. Given the relatively large number of candidate retinal features, LASSO regression was applied to reduce dimensionality and mitigate overfitting. The optimal penalty parameter (λ) was selected using the 1-standard error criterion (λ1se). Variables retained after LASSO selection were entered into multivariable logistic regression models. Age, sex, and body mass index (BMI) were included as predefined confounders regardless of statistical significance. Effect estimates are reported as odds ratios (ORs) with 95% confidence intervals (CIs). Model discrimination was evaluated using receiver operating characteristic (ROC) curves and the area under the curve (AUC) for the final multivariable model. All statistical tests were two-sided, and a *P*-value < 0.05 was considered statistically significant. No imputation was performed for missing data, and analyses were conducted using complete-case data only.

## 3 Results

### 3.1 Baseline characteristics

A total of 322 participants were included in the primary single-eye analysis, comprising 110 controls, 142 participants with CKD stages 1–2, and 70 participants with CKD stages 3–5. Baseline demographic and clinical characteristics are summarized in Table 1. Age differed significantly across groups, with the highest mean age observed in participants with CKD stages 3–5 (*P* = 0.024). In contrast, sex distribution, body mass index (BMI), intraocular pressure (IOP), spherical equivalent (SE), and best-corrected visual acuity (BCVA) did not differ significantly among groups.

**TABLE 1:** Sociodemographic and clinical characteristics of the study cohort.

Characteristics	Normal controls (*n* = 110)	CKD stages 1–2 (*n* = 142)	CKD stages 3–5 (*n* = 70)	*P*
Age, Mean ± SD, years	43.06 ± 12.38	40.04 ± 12.89	44.77 ± 12.43	0.024
Female, *n* (%)	67 (60.91)	73 (51.41)	32 (45.71)	0.112
BMI (kg/m^2^)	23.41 ± 3.53	24.09 ± 3.52	23.73 ± 3.22	0.292
IOP (mmHg)	15.42 ± 2.51	15.38 ± 3.08	14.89 ± 2.31	0.385
SE (D)	1.34 ± 1.46	1.65 ± 1.69	1.23 ± 1.45	0.127
BCVA	1.02 ± 0.13	1.01 ± 0.14	1.00 ± 0.14	0.727

Clinical characteristics of the CKD subgroup are summarized in Table 2. Among patients with CKD, hypertension was the most prevalent comorbidity (53.8%), followed by diabetes (10.4%). Cardiovascular disease was relatively uncommon (2.3%). A subset of patients received immunosuppressive therapy (11.8%) or hydroxychloroquine (3.8%).

**TABLE 2:** Clinical characteristics of participants with CKD.

Characteristics	Participants with CKD (*n* = 212)
Diabetes, *n* (%)	22 (10.38)
Hypertension, *n* (%)	114 (53.8)
Cardiovascular disease, *n* (%)	5 (2.3)
Hydroxychloroquine, *n* (%)	8 (3.8)
Immunosuppressants, *n* (%)	25 (11.8)

### 3.2 Differences in retinal microvascular parameters across groups

Group comparisons of retinal microvascular parameters are presented in Table 3. Several parameters differed significantly across groups. The AVR showed a progressive decrease from controls to CKD stages 1–2 and CKD stages 3–5 (0.65 ± 0.04, 0.64 ± 0.04, and 0.63 ± 0.03, respectively; *P* = 0.011). Arterial tortuosity (aTort) demonstrated a decreasing trend across groups, although this did not reach statistical significance (*P* = 0.052). Arterial vascular density (aVD) was lower in CKD compared with controls (*P* = 0.012), whereas venous vascular density (vVD) was higher in CKD (*P* = 0.001). Fractal dimension (FD) was also reduced in CKD groups (*P* = 0.002), indicating decreased vascular complexity.

**TABLE 3:** Comparison of retinal parameters among normal controls, CKD stages 1–2, and CKD stages 3–5.

Fundus parameters	Normal controls	CKD stages 1–2	CKD stages 3–5	*P*
AVR	0.65 ± 0.04	0.64 ± 0.04	0.63 ± 0.03	0.011
aFD	1.25 ± 0.04	1.25 ± 0.05	1.25 ± 0.05	0.151
vFD	1.01 ± 0.09	1.03 ± 0.07	1.01 ± 0.08	0.011
aTort	0.67 ± 0.12	0.65 ± 0.09	0.63 ± 0.10	0.052
vTort	0.81 ± 0.09	0.85 ± 0.12	0.83 ± 0.09	0.011
aDWA	71.64 ± 7.00	69.83 ± 6.54	69.93 ± 5.25	0.065
vDWA	110.04 ± 11.35	108.98 ± 7.67	110.47 ± 7.87	0.468
aVD	0.03 ± 0.01	0.03 ± 0.01	0.03 ± 0.01	0.012
vVD	0.06 ± 0.01	0.06 ± 0.01	0.06 ± 0.01	0.001
FD	1.10 ± 0.06	1.11 ± 0.07	1.08 ± 0.06	0.002
VDiam	92.23 ± 10.42	90.99 ± 9.18	93.43 ± 6.99	0.182
VTort	0.72 ± 0.07	0.74 ± 0.07	0.72 ± 0.06	0.027
DMA	7.42 ± 3.73	7.35 ± 3.75	6.54 ± 3.71	0.252
DAR	1.15 ± 0.09	1.15 ± 0.09	1.14 ± 0.09	0.756
MCD	17762.30 ± 2329.76	18168.25 ± 2454.72	18757.30 ± 2451.74	0.027
VDWFI	0.05 ± 0.01	0.06 ± 0.01	0.05 ± 0.01	<0.001
VL	1478.91 ± 220.35	1417.78 ± 192.19	1535.94 ± 206.53	<0.001
FD3PDTS	1.11 ± 0.07	1.09 ± 0.07	1.08 ± 0.06	0.054
VT3PD	0.83 ± 0.10	0.86 ± 0.10	0.85 ± 0.11	0.179
VDiam3PD	100.59 ± 9.86	100.04 ± 9.05	102.05 ± 7.74	0.316

AVR, arteriovenous ratio; aFD, arterial fractal dimension; vFD, venous fractal dimension; aTort, arterial tortuosity; vTort, venous tortuosity; aDWA, arterial diameter (wide-angle); vDWA, venous diameter (wide-angle); aVD, arterial vessel density; vVD, venous vessel density; FD, fractal dimension; VDiam, vessel diameter; VTort, vessel tortuosity; DMA, disk–macula angle; DAR, disk axis ratio; MCD, macular center-to-boundary distance; VDWFI, vessel density weighted fractal index; VL, vessel length; FD3PDTS, fractal dimension in the superior-temporal region within the 3PD area; VT3PD, vessel tortuosity within the 3PD region; VDiam3PD, vessel diameter within the 3PD region.

In addition, venous tortuosity (vTort) and vascular length (VL) differed significantly across groups (*P* = 0.011 and *P* < 0.001, respectively). The venous-derived parameter VDWFI also showed a significant difference (*P* < 0.001). Other parameters, including venous diameter (wide-angle) (vDWA), vessel diameter (VDiam), disk–macula angle (DMA), disk axis ratio (DAR), and vessel tortuosity within the 3PD region (VT3PD), did not show statistically significant differences across groups (all *P* > 0.05). Overall, the observed changes were generally consistent across CKD stages, with more pronounced alterations in stages 3–5.

### 3.3 Feature selection, multivariable analysis, and model performance

To reduce feature dimensionality and mitigate overfitting, feature selection was performed using least absolute shrinkage and selection operator (LASSO) regression with 10-fold cross-validation (Supplementary Figures 1, 2). The optimal penalty parameter (λ) was selected using the 1-standard error criterion (λ1se), resulting in a parsimonious set of predictors. The variables retained at λ1se included AVR, aTort, aVD, vVD, and VT3PD, along with demographic covariates (age, sex, and BMI). These variables were subsequently entered into multivariable logistic regression models (Table 4).

**TABLE 4:** Multivariable logistic regression analysis of selected retinal parameters associated with CKD.

Fundus parameters	OR	95% CI	*P*
Age	0.806	0.578–1.119	0.199
Sex	1.175	0.672–2.056	0.572
BMI	1.078	0.814–1.429	0.600
AVR	0.617	0.433–0.866	0.006
aTort	0.380	0.230–0.609	<0.001
aVD	0.642	0.430–0.948	0.027
vVD	1.910	1.327–2.798	<0.001
VT3PD	2.020	1.186–3.540	0.012

**FIGURE 1:**
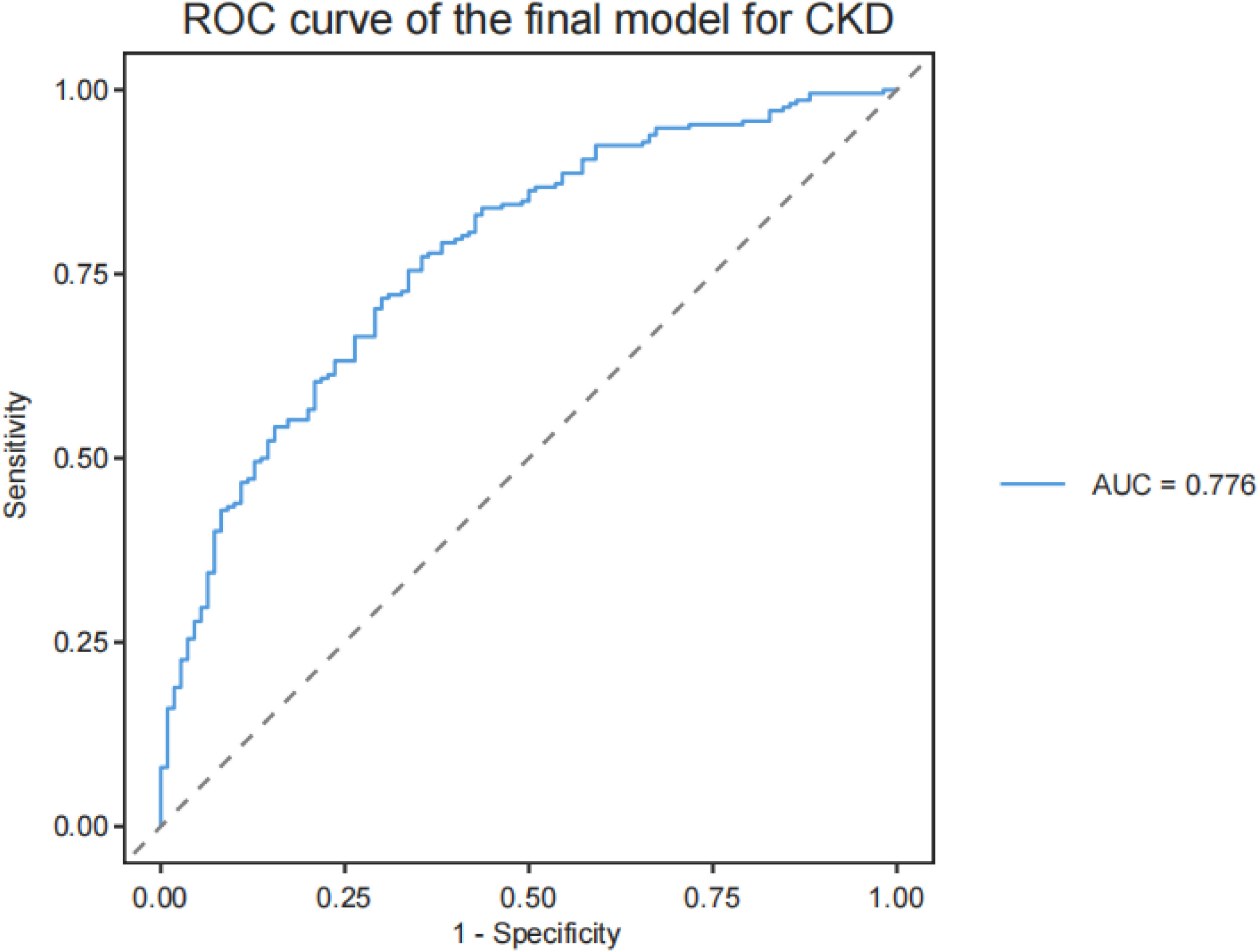
Receiver operating characteristic (ROC) curve of the final multivariable model for discriminating CKD from controls. The model demonstrated moderate discrimination (AUC = 0.776).

**FIGURE 2:**
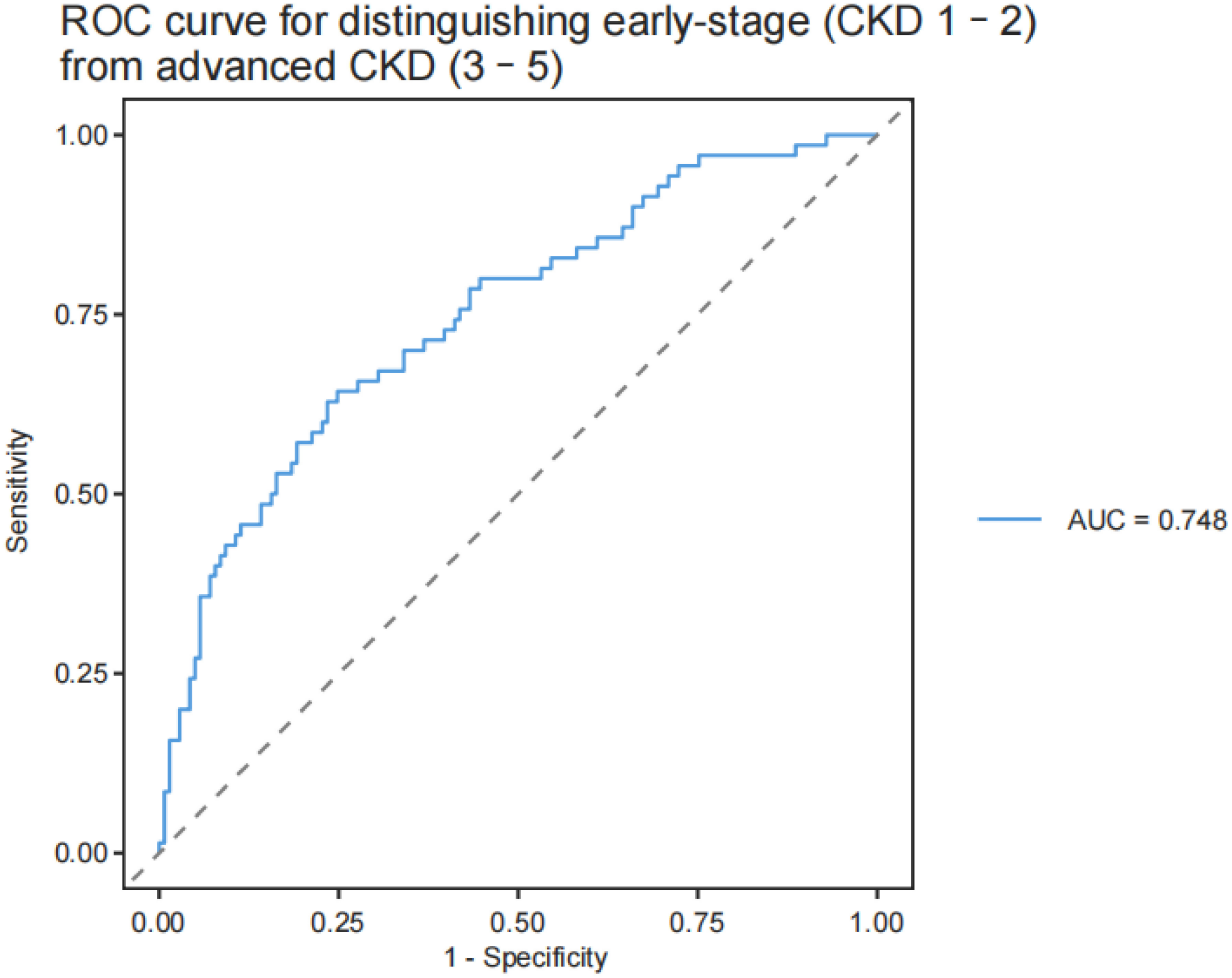
Receiver operating characteristic (ROC) curve for distinguishing early-stage CKD (stages 1–2) from more advanced CKD (stages 3–5). The model showed modest discrimination (AUC = 0.748).

After adjustment for age, sex, and BMI, lower AVR was significantly associated with higher odds of CKD (OR = 0.617, 95% CI: 0.433–0.866, *P* = 0.006). Reduced arterial tortuosity (aTort) was also independently associated with CKD (OR = 0.380, 95% CI: 0.230–0.609, *P* < 0.001), as was lower arterial vascular density (aVD, OR = 0.642, 95% CI: 0.430–0.948, *P* = 0.027). In contrast, higher venous vascular density (vVD) was positively associated with CKD (OR = 1.910, 95% CI: 1.327–2.798, *P* < 0.001). The parameter VT3PD was also independently associated with CKD (OR = 2.020, 95% CI: 1.186–3.540, *P* = 0.012). Model discrimination was evaluated using receiver operating characteristic (ROC) analysis. The final model demonstrated moderate discrimination for CKD, with an area under the curve (AUC) of 0.776 (Figure 1). In an exploratory analysis, the model showed limited ability to distinguish between early-stage (CKD stages 1–2) and more advanced CKD (stages 3–5), with an AUC of 0.748 (Figure 2).

## 4 Discussion

In this case-control study, we identified a limited set of AI-derived retinal microvascular parameters that were associated with CKD after adjustment for major confounders. Lower AVR, reduced aTort, and lower aVD were inversely associated with CKD, whereas higher vVD and VT3PD showed positive associations. These findings suggest that retinal microvascular alterations are associated with CKD and may reflect systemic microvascular changes.

The observed pattern of decreased AVR, reduced aTort, and lower aVD, together with increased venous density, is consistent with microvascular alterations reported in CKD. These changes may reflect endothelial dysfunction, vascular rarefaction, and altered hemodynamics, which have been described in both renal and retinal circulations. In this context, the retinal vasculature may serve as an accessible surrogate for systemic microvascular status (21).

Several retinal parameters were already altered in early CKD (stages 1–2), and the direction of these changes was generally consistent across disease stages. Previous studies have primarily focused on advanced CKD (stages 3–5), whereas early-stage CKD has been less frequently investigated (22). These findings indicate that retinal microvascular alterations may already be detectable in early stages of CKD, highlighting their potential as early, non-invasive biomarkers of disease-related microvascular changes (14). Although this study was not designed to formally assess disease progression, stage-based comparisons provide supportive evidence for this interpretation.

The final model demonstrated moderate discrimination for CKD (AUC = 0.776). This level of performance suggests that retinal biomarkers alone are unlikely to serve as standalone diagnostic tools but may provide complementary, non-invasive information for CKD risk identification. Given that the model was based primarily on fundus-derived variables and basic demographic factors, the observed discrimination may be considered hypothesis-generating (23, 24).

Several methodological aspects support the robustness of the present analysis. First, to address inter-eye dependency, the primary analysis was restricted to one eye per participant. Second, feature dimensionality was reduced using LASSO regression, enabling identification of a parsimonious set of retinal parameters. Third, multivariable models were applied to adjust for key confounders, improving the interpretability of the findings (25).

This study has several limitations. First, the cross-sectional design precludes causal inference. Second, although the primary analysis was restricted to one eye per participant to reduce inter-eye correlation, this approach may reduce statistical efficiency and does not fully capture bilateral variability. Third, the study was conducted at a single center, which may limit generalizability. In addition, the sample size for advanced CKD (stages 3–5) was relatively limited, which may reduce the stability of stage-specific comparisons. Fourth, although major confounders such as age, sex, and BMI were adjusted for, other important clinical factors, including hypertension, diabetes, and medication use, were not included in the multivariable models to avoid overfitting; therefore, residual confounding cannot be excluded. Fifth, although image quality control procedures were applied, unmeasured factors such as mild-to-moderate media opacity (e.g., early cataract) may have influenced retinal image quality and quantitative measurements. Finally, the model demonstrated only moderate discrimination and requires external validation in independent and more diverse populations before potential clinical application (26).

In conclusion, a limited set of AI-derived retinal microvascular parameters was associated with CKD and demonstrated moderate discriminatory ability. Importantly, several parameters were already altered in early-stage CKD, suggesting that retinal microvascular changes may serve as early, non-invasive indicators of disease-related microvascular dysfunction. These findings support the potential of fundus image–derived biomarkers for CKD risk identification. Further validation in larger, multi-center cohorts is warranted (27, 28).

## Data Availability

The original contributions presented in this study are included in this article/Supplementary material, further inquiries can be directed to the corresponding author.
