# Supplementary material for: Artificial intelligence-based quantification of retinal microvascular biomarkers from fundus photography of chronic kidney disease: a case-control study
Source: Front Med (Lausanne). 2026 May 18;13:1719984. doi: 10.3389/fmed.2026.1719984 (PMC13222801; doi:10.3389/fmed.2026.1719984)
Supplement: Supplementary file 1 [file Data_Sheet_1.docx]

**Feature selection was performed using LASSO regression (Supplementary Figure S1–S2).**


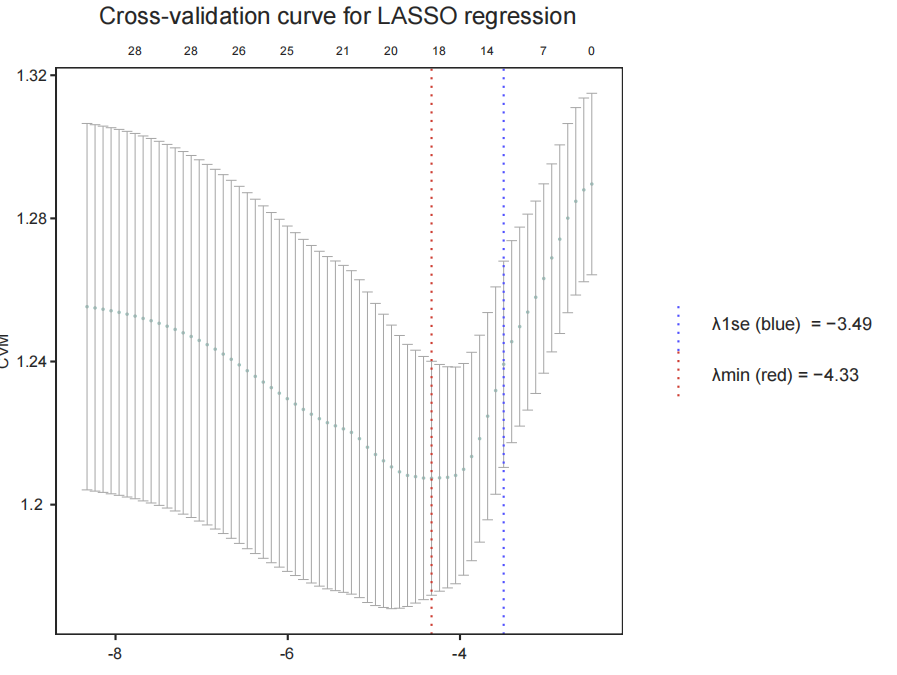


**Supplementary Figure S1**. LASSO cross-validation curve for selection of the optimal penalty parameter (λ). The mean cross-validated error is shown with standard error bars. The vertical dashed lines indicate the minimum error (λmin) and the 1-standard error criterion (λ1se).


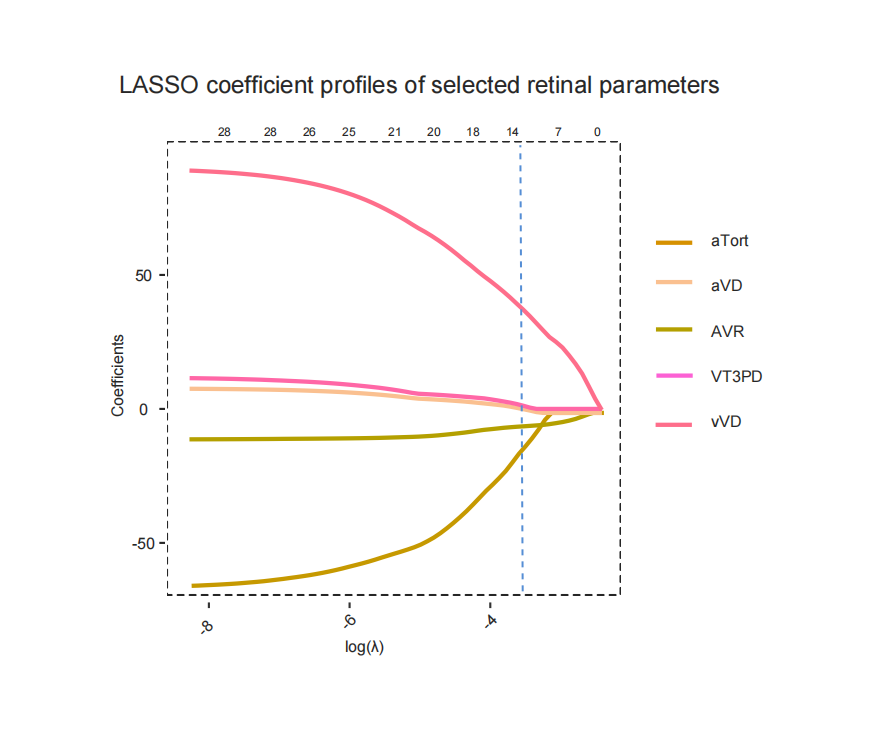


**Supplementary Figure S2**. LASSO coefficient profiles of retinal parameters. Each curve represents the trajectory of a variable coefficient as a function of log(λ). As λ increases, coefficients shrink toward zero, and less informative variables are eliminated.
